# From Disease Control to Long-Term Stability: Regenerative and Prosthetic Management of Peri-Implantitis—A Five-Year Case Report

**DOI:** 10.3390/reports9030270

**Published:** 2026-08-13

**Authors:** Jakub Kwiatek, Oskar Barczak, Marcin Lenkowski, Justyna Kaczewiak, Mateusz Tarnowski

**Affiliations:** 1 Kwiatek Dental Clinic, Kordeckiego 22, 60-144 Poznań, Poland; 2 Wojskowa Specjalistyczna Przychodnia Lekarska, Poradnia Chirurgii Stomatologicznej, ul. Szylinga 1, 61-787 Poznań, Poland

**Keywords:** peri-implantitis, bone regeneration, platelet-rich fibrin, emergence profile, case report

## Abstract

**Background and Clinical Significance**: Peri-implantitis is an inflammatory condition associated, among other factors, with biofilm accumulation, affecting peri-implant soft and hard tissues and potentially leading to implant loss. Its treatment remains challenging because no single decontamination or regenerative protocol has demonstrated clear superiority. This case report describes a comprehensive surgical and regenerative approach aimed at preserving an affected implant and restoring peri-implant tissue stability; **Case Presentation**: A systemically healthy 30-year-old patient presented with peri-implant bone loss around an implant in position 25, restored with a lithium disilicate crown and functioning for three years. Treatment included flap elevation, mechanical debridement and air-polishing of the implant surface, followed by thorough irrigation with sterile saline to remove residual abrasive particles and debris, photodynamic antimicrobial therapy, and laser therapy. Bone regeneration was performed using a bone substitute combined with injectable platelet-rich fibrin to produce sticky bone, which was covered with an advanced platelet-rich fibrin membrane. A provisional crown was placed without occlusal contact. After four months, a definitive crown with a modified emergence profile was delivered to improve hygienic access and reduce biofilm retention. Clinical and radiographic follow-up over five years demonstrated stable peri-implant tissues and maintained bone levels; **Conclusions**: The combined use of surgical decontamination, PRF-assisted regeneration, sticky bone, and prosthetic modification resulted in stable clinical and radiographic outcomes over five years. Identification and elimination of contributing factors were essential for long-term treatment success.

## 1. Introduction and Clinical Significance

Dental implants are considered the gold standard for the rehabilitation of missing teeth. The outcome of implant–prosthetic treatment depends on a number of factors, including genetic and immunological conditions, concomitant systemic diseases, and the quality and quantity of bone tissue [1], together with the operator’s experience and the appropriate planning and performance of the surgical procedure.

The oral cavity is inhabited by a complex microbiota capable of colonising the surfaces of the teeth, tongue, and oral mucosa, as well as components of the implant system exposed to the oral environment [2]. Colonisation of the implant surface by bacteria can occur within as little as 30 min following implant placement [3]. This process is particularly important in relation to surfaces or components of the implant system exposed to the oral environment, such as the healing screw, prosthetic abutment, or crown. In the case of submerged healing, however, the intraosseous portion of the implant remains covered by tissues and is not directly exposed to contact with the oral microbiota. Initially, the microflora is usually less complex and consists predominantly of early colonising bacteria typical of the oral environment. Over time, the biofilm matures, and its composition becomes increasingly diverse. After approximately three months, the bacterial flora surrounding the implant may already resemble that observed around natural teeth. Under healthy peri-implant tissue conditions, the microbiota in the implant region consists mainly of bacteria belonging to the yellow complex, such as Streptococcus—Streptococcus oralis and Streptococcus mutans—as well as the purple complex, which includes, among others, Veillonella parvula and Actinomyces odontolyticus, representing early colonisers [4].

Under physiological conditions, commensal and potentially pathogenic microorganisms remain in a dynamic equilibrium maintained by an appropriate host response. Disruption of this equilibrium, particularly in the course of systemic diseases, impaired immunity, or inadequate oral hygiene, may lead to dysbiosis, defined as the predominance of microorganisms with greater pathogenic potential. Such a shift in the microbiota promotes the development of inflammation within the peri-implant tissues [5]. Oral dysbiosis should therefore not be regarded solely as a microbiological disturbance, but also as a clinically relevant condition that may lead to the development of pathological changes within the oral tissues. Importantly, this process may develop asymptomatically, without evident signs of inflammation, thereby delaying its diagnosis and the implementation of appropriate management [6].

Consequently, with the increasing prevalence of implant treatment, peri-implant tissue infections have become an important issue in oral pathology. They are classified as peri-implant mucositis, defined as inflammation of the mucosa surrounding an implant, and peri-implantitis, defined as inflammation of the peri-implant tissues accompanied by progressive bone loss. In addition to the presence of bacterial biofilm, risk factors for these conditions include poorly fitting or improperly designed fixed prosthetic restorations, cement-retained restorations, and a history of periodontitis [7]. Peri-implantitis is a chronic condition that leads to the destruction and loss of bone tissue, consequently altering its morphology and potentially resulting in the loss of the dental implant [8].

Various decontamination methods are used in the treatment of peri-implantitis, including mechanical, chemical, and laser-based approaches. These may additionally be combined with bone and soft tissue regeneration procedures. Their common objective is to eliminate bacterial biofilm from the peri-implant region, reduce inflammation, and create conditions conducive to bone tissue regeneration [9]. However, there is currently no conclusive evidence demonstrating the superiority of one decontamination method over the others, and consequently, no standardised treatment protocol for peri-implantitis has been established [10].

Platelet-rich fibrin (PRF), introduced by Choukroun et al. in 2001 [11], is a second-generation platelet concentrate obtained from the patient’s blood without the addition of anticoagulants or biochemical modification. It consists of a fibrin matrix containing platelets, leukocytes, stem cells, and cytokines. Growth factors are gradually released from this matrix, stimulating the proliferation of osteoblasts and fibroblasts, supporting angiogenesis, and accelerating tissue healing. Due to its ability to promote the healing of both soft and hard tissues, PRF has found broad application in periodontology, surgery, and implantology, including the treatment of gingival recessions, bone defects, and peri-implant defects. When combined with bone substitute materials, it may support bone regeneration, improve healing conditions, and promote tissue reconstruction around implants [12].

The aim of this case report was to present the outcomes of peri-implantitis treatment using surgical and regenerative procedures to restore an adequate amount of bone around the implant.

## 2. Case Presentation

**Figure 1:**
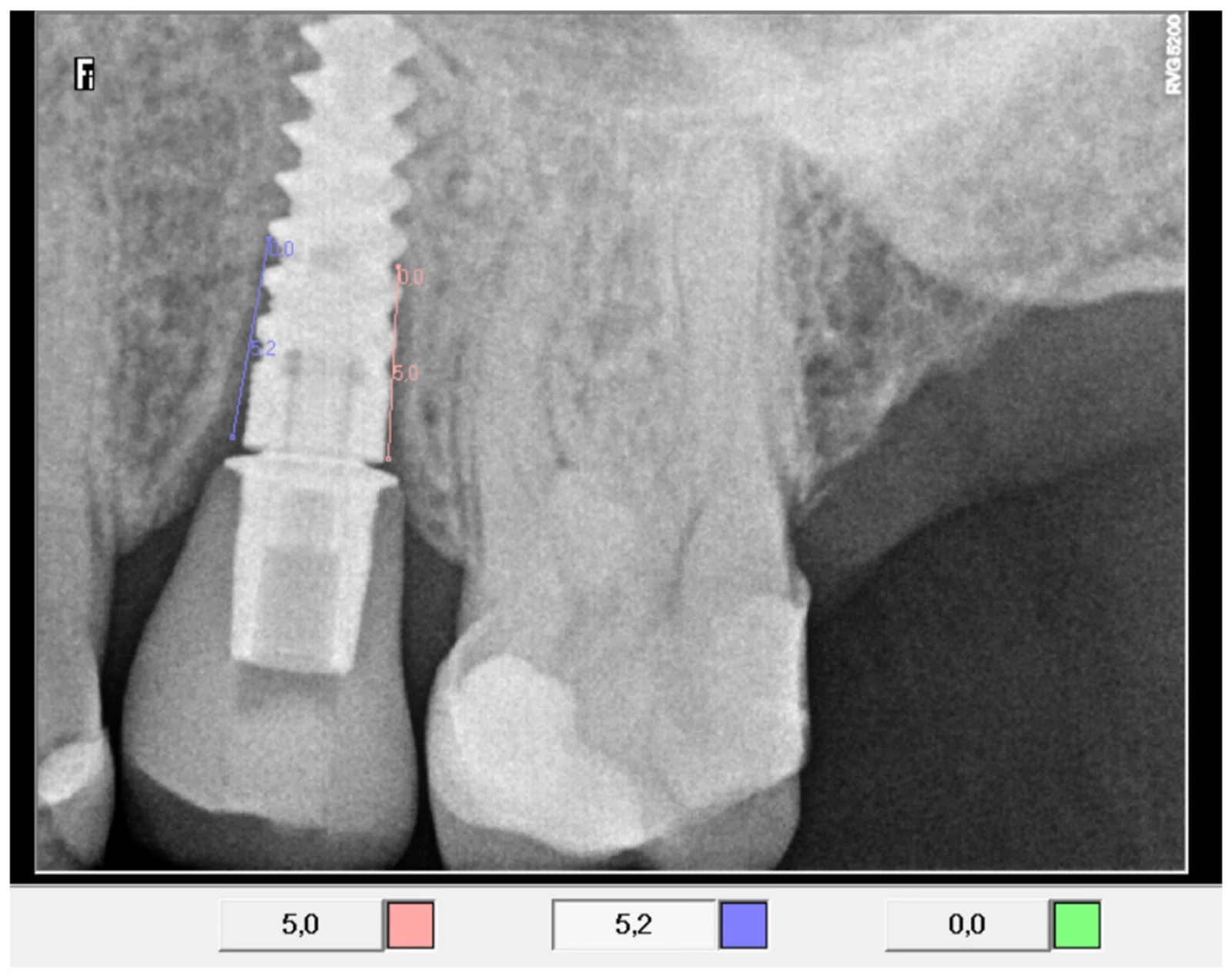
Radiograph of the implant in position 25 obtained at the initial follow-up visit.

**Figure 2:**
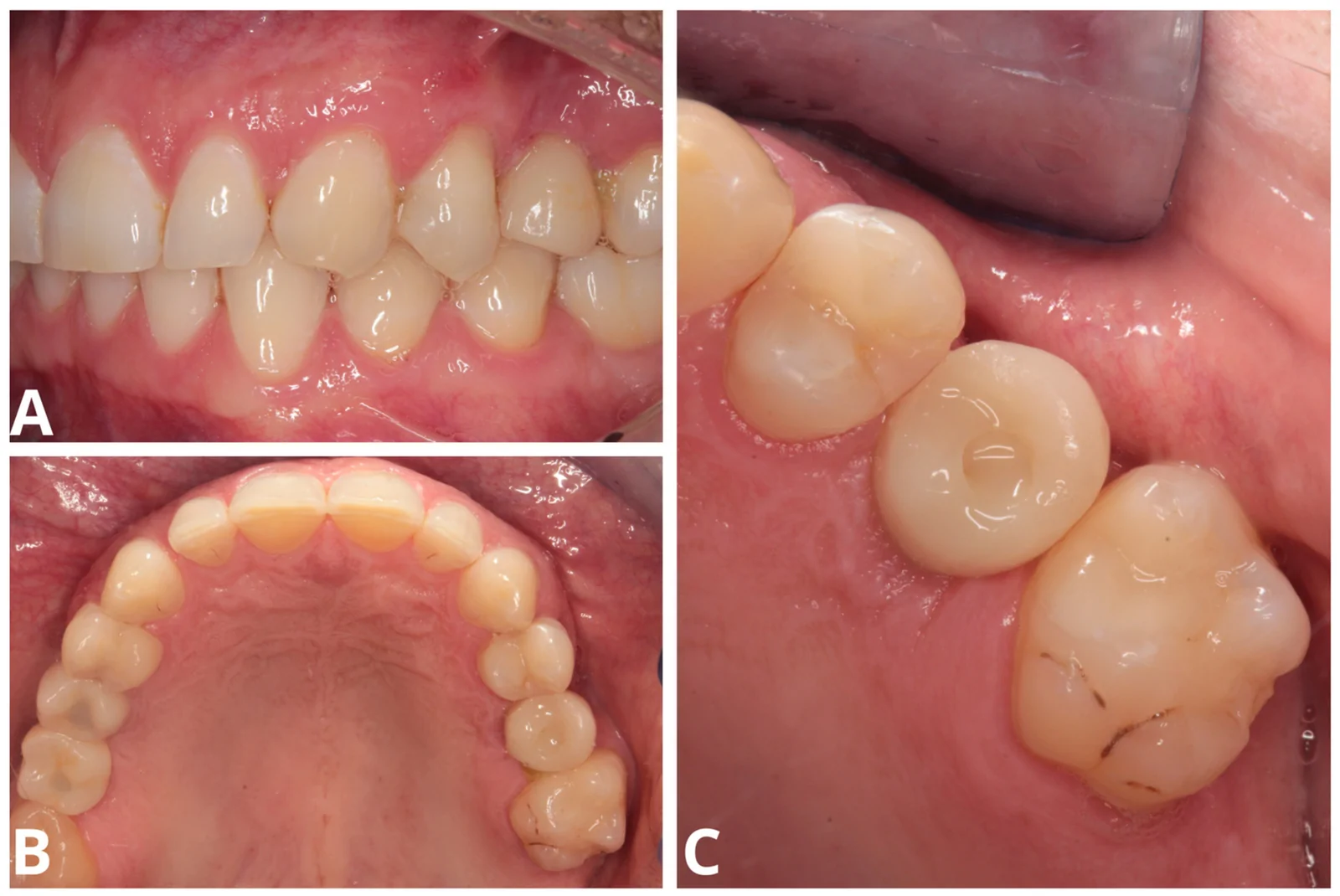
Baseline clinical situation: (**A**)—left lateral view in occlusion; (**B**)—occlusal view of the maxillary arch; (**C**)—occlusal view of the prosthetic crown in position 25.

A 30-year-old systemically healthy, asymptomatic patient with no history of regular medication use presented with a bone-level dental implant placed at site 25 (ALPHA BIO NEO (Alpha-Bio Tec Ltd., Petah Tikva, Israel), 3.5 × 11.5 mm), restored with a lithium disilicate crown that had been in function for three years. Radiographic assessment performed during a follow-up examination (Figure 1) revealed peri-implant bone loss consistent with a diagnosis of peri-implantitis. Vertical bone loss was observed on both the mesial and distal aspects of the implant. The vertical defect depth, measured from the implant platform to the first radiographically visible bone-to-implant contact, was 5.2 mm and 5.0 mm, respectively. Based on the clinical and radiographic findings, bone augmentation and replacement of the existing implant-supported crown were indicated. For the purpose of designing the new prosthetic restoration, an intraoral digital impression was obtained using a scan post. The baseline clinical condition is presented in Figure 2. The sequence of treatment stages is summarized in Table 1.

Prior to the surgical procedure, antibiotic prophylaxis was administered in accordance with Polish recommendations permitting its consideration in implant procedures combined with bone augmentation. The patient received two Augmentin 1000 mg tablets one hour before the procedure. Each tablet contained 875 mg of amoxicillin and 125 mg of clavulanic acid, resulting in a total dose of 1750 mg of amoxicillin and 250 mg of clavulanic acid. The patient also received ibuprofen (400 mg), a non-steroidal anti-inflammatory drug (NSAID), to control perioperative pain and inflammation.

**Figure 3:**
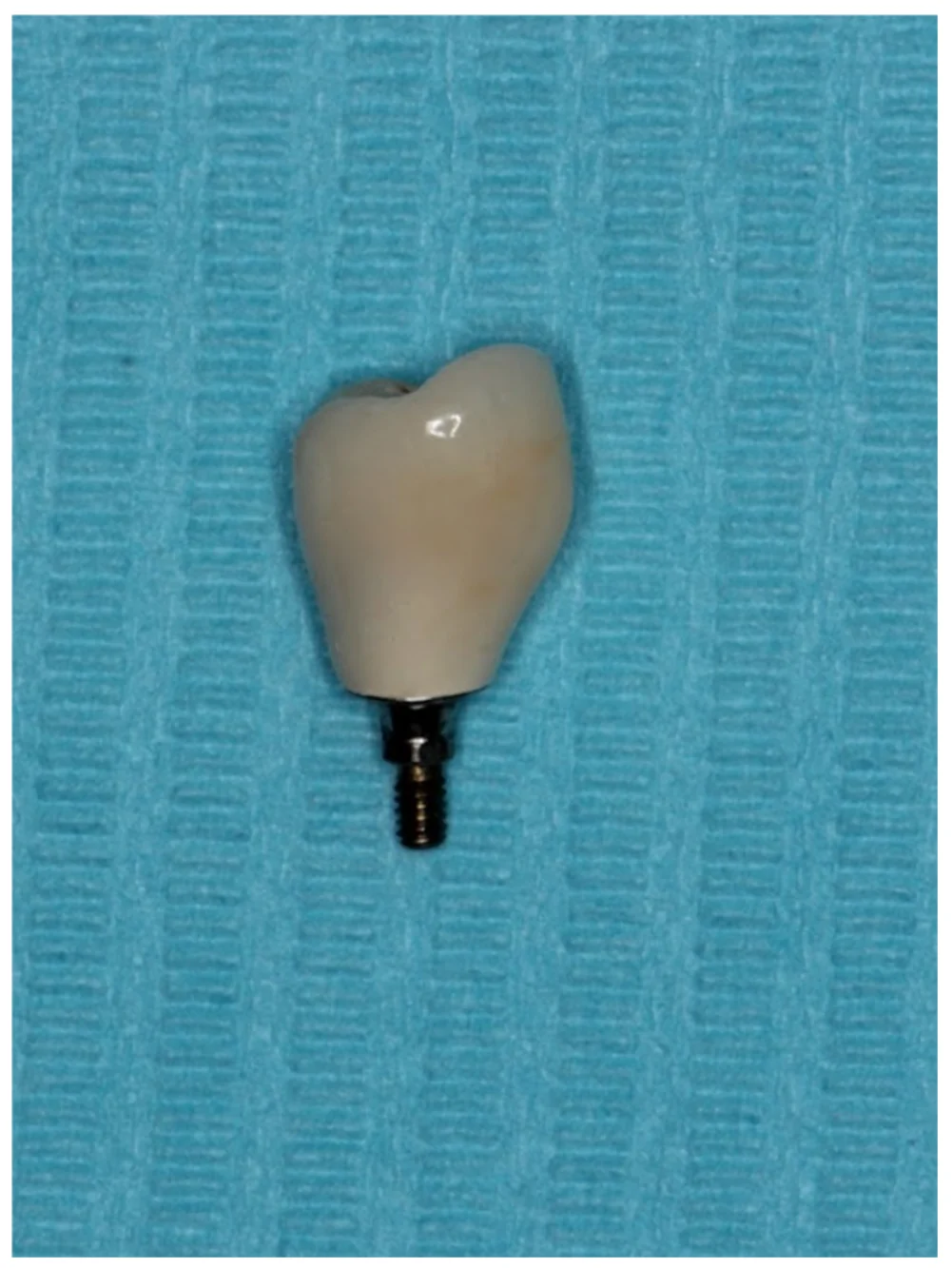
Removed prosthetic crown in position 25.

**Figure 4:**
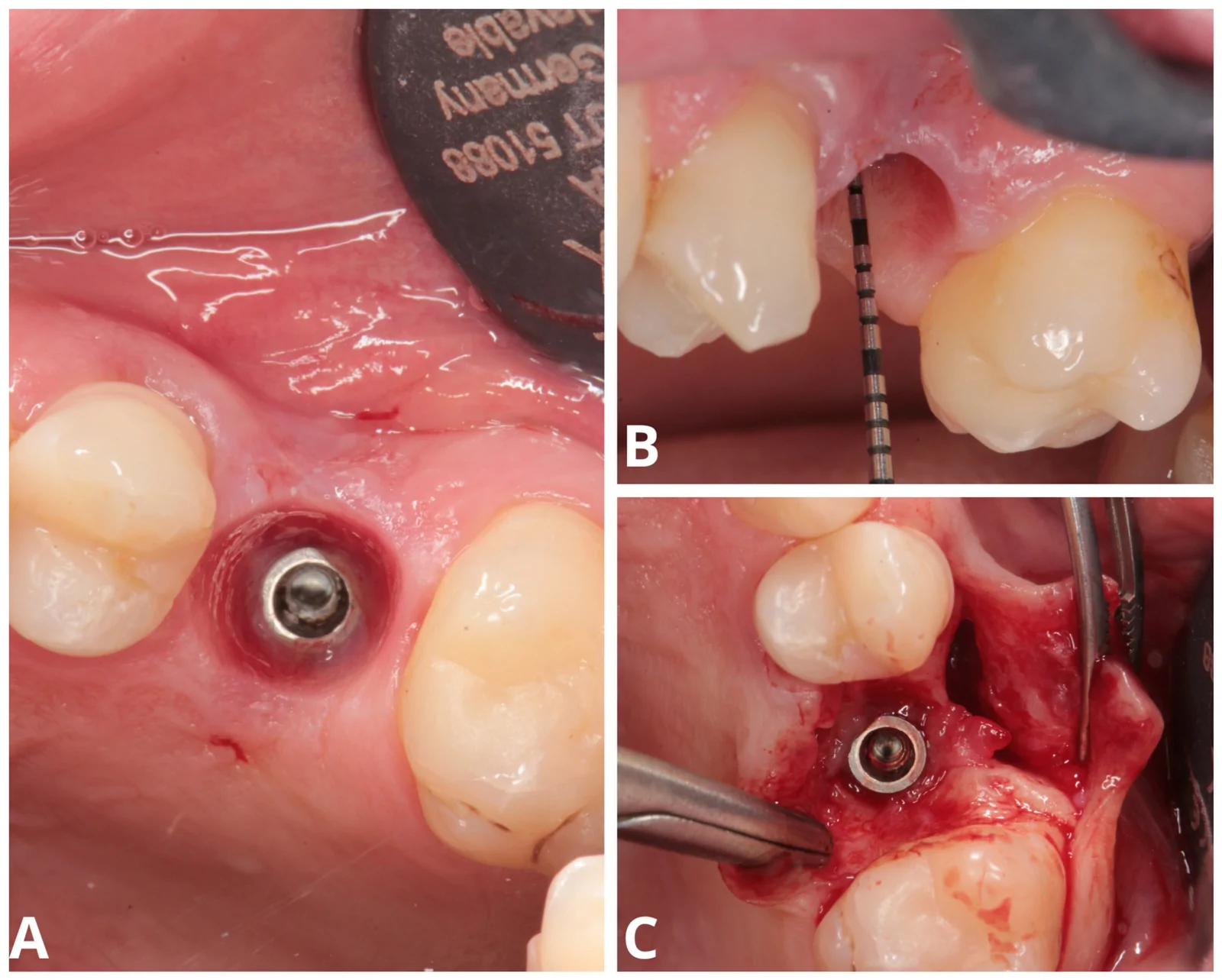
Clinical and surgical assessment of the peri-implant site: (**A**)—occlusal view following removal of the prosthetic crown; (**B**)—measurement of the probing pocket depth; (**C**)—elevation of the mucoperiosteal flap prior to soft tissue debridement.

The prosthetic crown in position 25 was then removed (Figure 3), and the peri-implant probing depth was assessed. Probing depths ranged from 4.5 to 7.0 mm and were measured at four sites around the implant: buccal, palatal, mesial, and distal. The maximum probing depth of 7.0 mm was recorded at the mesial site. Bleeding on probing was observed, while the amount of plaque was minimal. Local infiltration anaesthesia was achieved using 2% lidocaine with epinephrine at a concentration of 1:80,000. A mucoperiosteal flap was subsequently elevated to provide access to the osseous defect (Figure 4).

**Figure 5:**
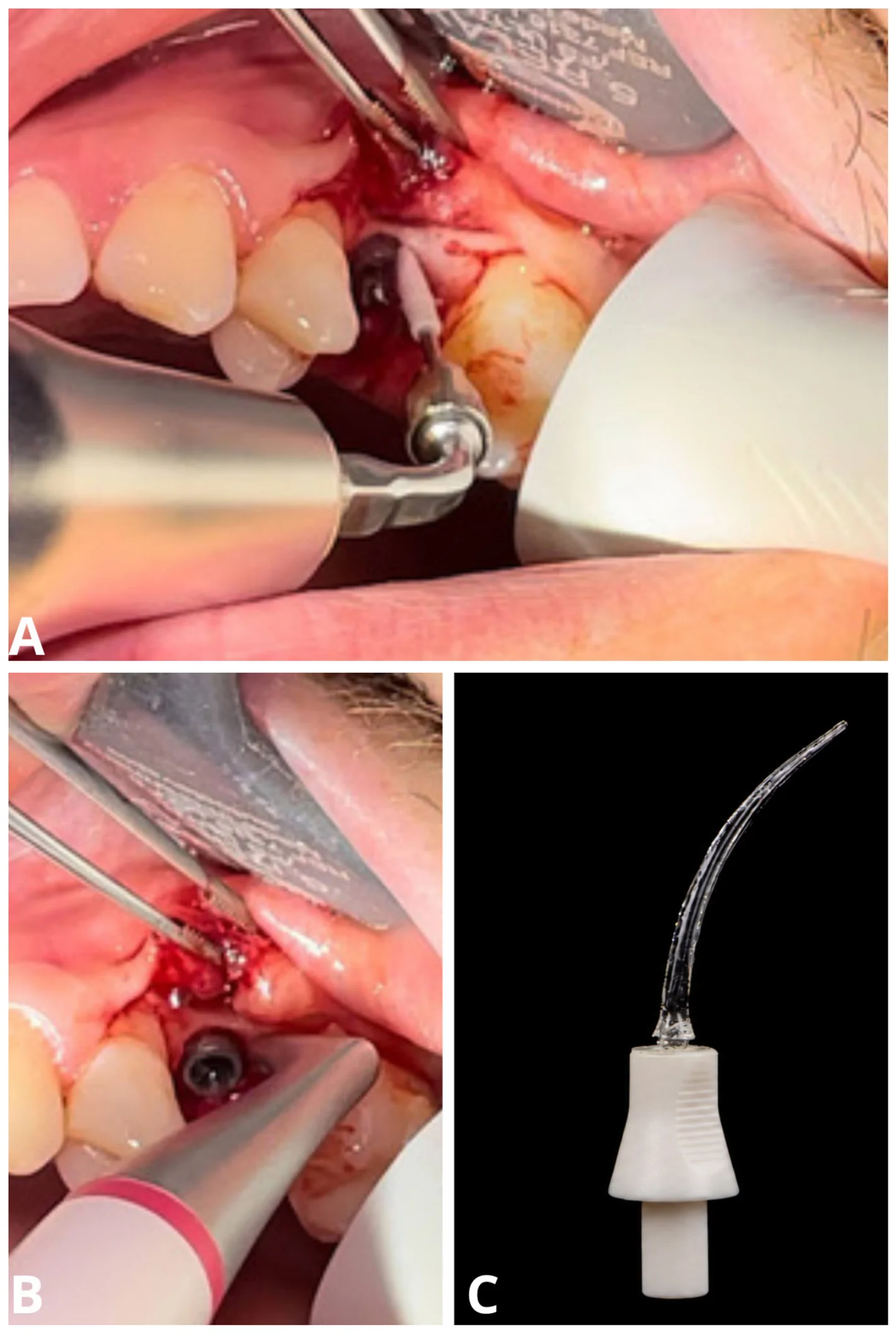
Implant surface decontamination: (**A**)—scaling; (**B**)—air polishing; (**C**)—Lasotronix laser tip used for photoactivated disinfection.

The next stage involved mechanical and chemical decontamination of the implant surface (Figure 5). Peri-implant scaling was performed using dedicated EMS Instrument Piezon PI MAX Starter Kit tips (reference FS-295; EMS Electro Medical Systems S.A., Nyon, Switzerland) composed of polyether ether ketone reinforced with 30% carbon fibres. These instruments are designed for the safe debridement of implant surfaces without damaging the titanium surface. The procedure included the use of an ultrasonic scaler to remove any mineralised biofilm deposits and mechanically disrupt the biofilm structure. In addition, cavitation and microstreaming generated during operation of the device may contribute to bacterial cell damage and a reduction in the microbial load [13,14]. The specific design of these tips not only minimises the risk of scratching the implant surface but may also contribute to limiting the progression of peri-implant disease [15]. Air polishing was subsequently performed using the EMS Airflow Prophylaxis Master device (reference FT-229/A; EMS Electro Medical Systems S.A., Nyon, Switzerland) and PLUS powder (reference DV-082; EMS Electro Medical Systems S.A., Nyon, Switzerland), an erythritol-based powder with a particle size of 14 μm. Erythritol has been described as a beneficial adjunct in bacterial biofilm control and in the prevention of dental caries and periodontal disease. Owing to its very fine particle size, this powder enables atraumatic removal of bacterial biofilm from implant surfaces and peri-implant tissues while maintaining the safety of delicate titanium structures and soft tissues [16]. The decontamination procedure was completed by thorough irrigation of the peri-implant region with sterile saline to remove residual abrasive particles and debris following implant surface decontamination. This was followed by photodynamic antimicrobial therapy (PAD) using a Lasertronix laser system (Lasotronix, Poland) at a wavelength of 635 nm, with the aim of achieving an additional reduction in the microbial load within the treated area [17]. Laser biostimulation was subsequently performed using the same wavelength to support tissue healing and regeneration [18].

Platelet-rich fibrin was prepared from the patient’s previously collected blood in two forms: injectable platelet-rich fibrin (i-PRF) and advanced platelet-rich fibrin (A-PRF). For i-PRF and A-PRF preparation, two tubes of the patient’s venous blood were centrifuged simultaneously at 1300 rpm for 14 min in accordance with the established protocol [19]. Both preparations were used during the subsequent augmentation procedure.

**Figure 6:**
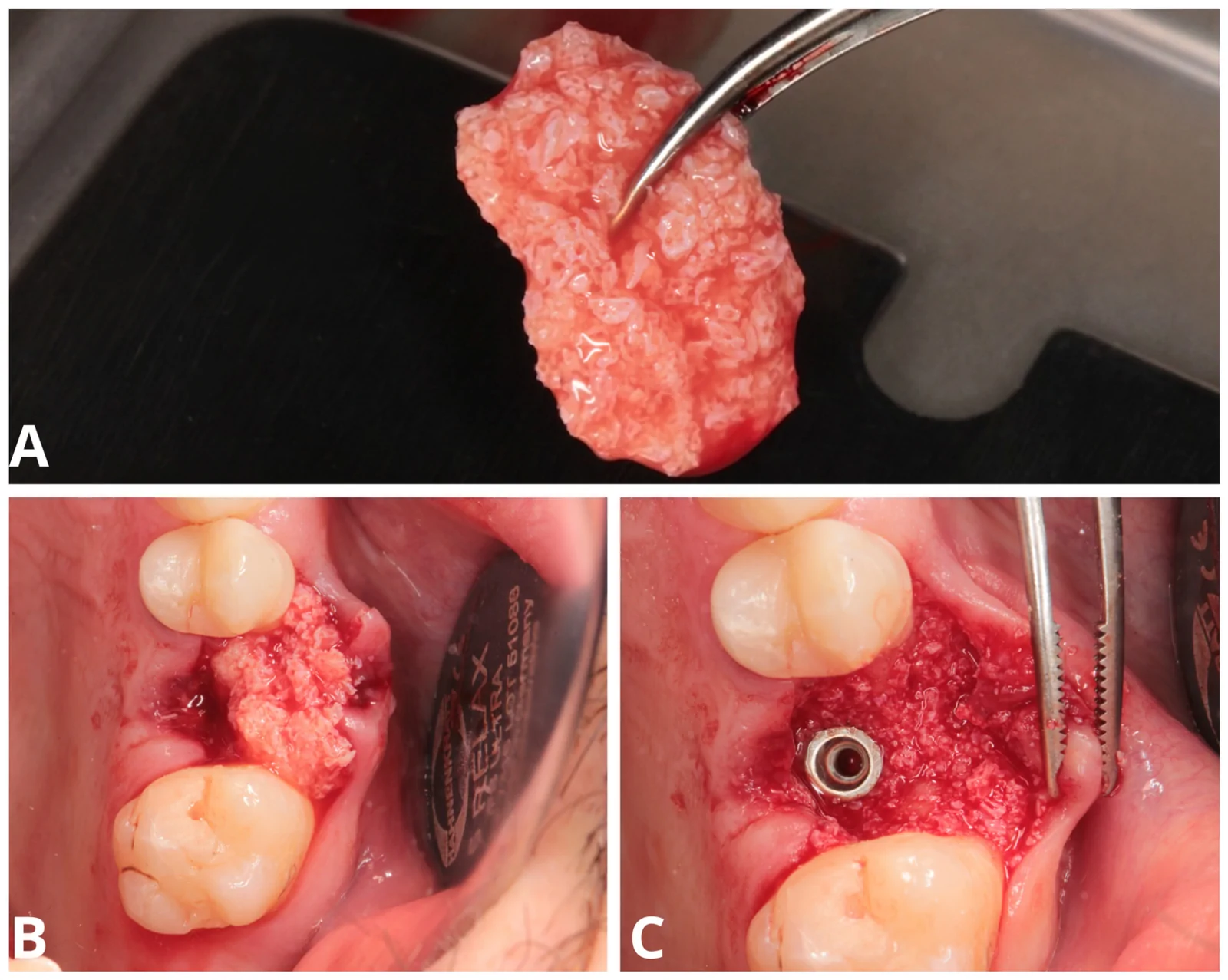
Preparation of the sticky bone material: (**A**)—shaped sticky bone material; (**B**)—sticky bone material placed within the osseous defect; (**C**)—view of the sticky bone material following adaptation to the augmentation site.

**Figure 7:**
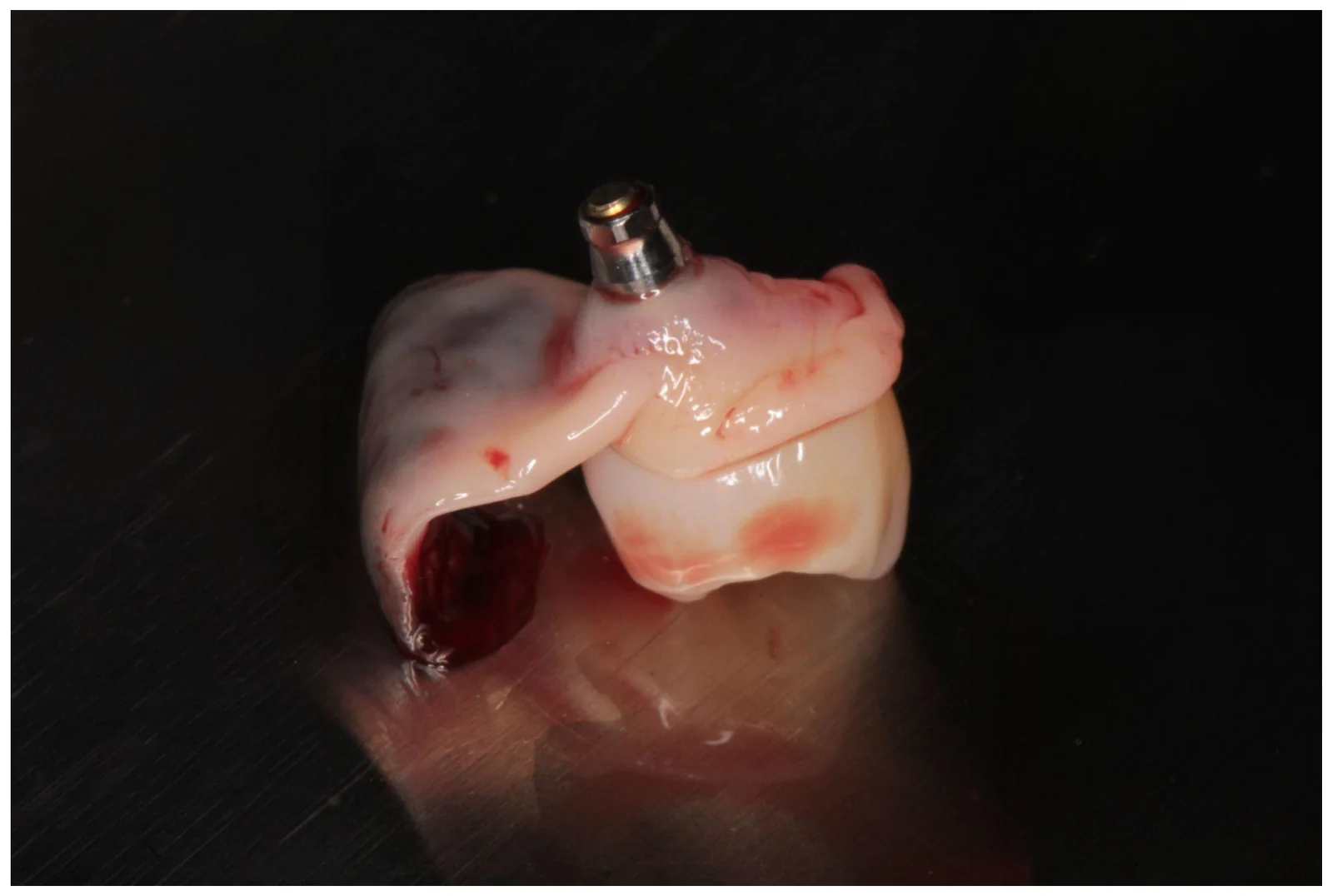
PRF membrane positioned over the new provisional crown.

Bone augmentation was performed using 0.5 g of The Graft (catalog no. BG-A05; Purgo Biologics Inc., Seongnam, Republic of Korea) bone substitute material combined with i-PRF to obtain so-called “sticky bone” (Figure 6). The graft particles were first moistened with autologous blood collected during A-PRF preparation and then gently combined with freshly prepared liquid i-PRF until a cohesive and moldable consistency was obtained. The resulting composite graft was used immediately after preparation [20]. Owing to its plasticity, the material could be precisely adapted to the morphology of the defect, enabling complete filling of the osseous deficiency and stabilisation of the bone substitute. This, in turn, was intended to maximise the likelihood of successful bone regeneration in the area affected by bone loss [21]. An A-PRF membrane was prepared (Figure 7) to maintain the augmented material in the desired position and support the healing process [22].

**Figure 8:**
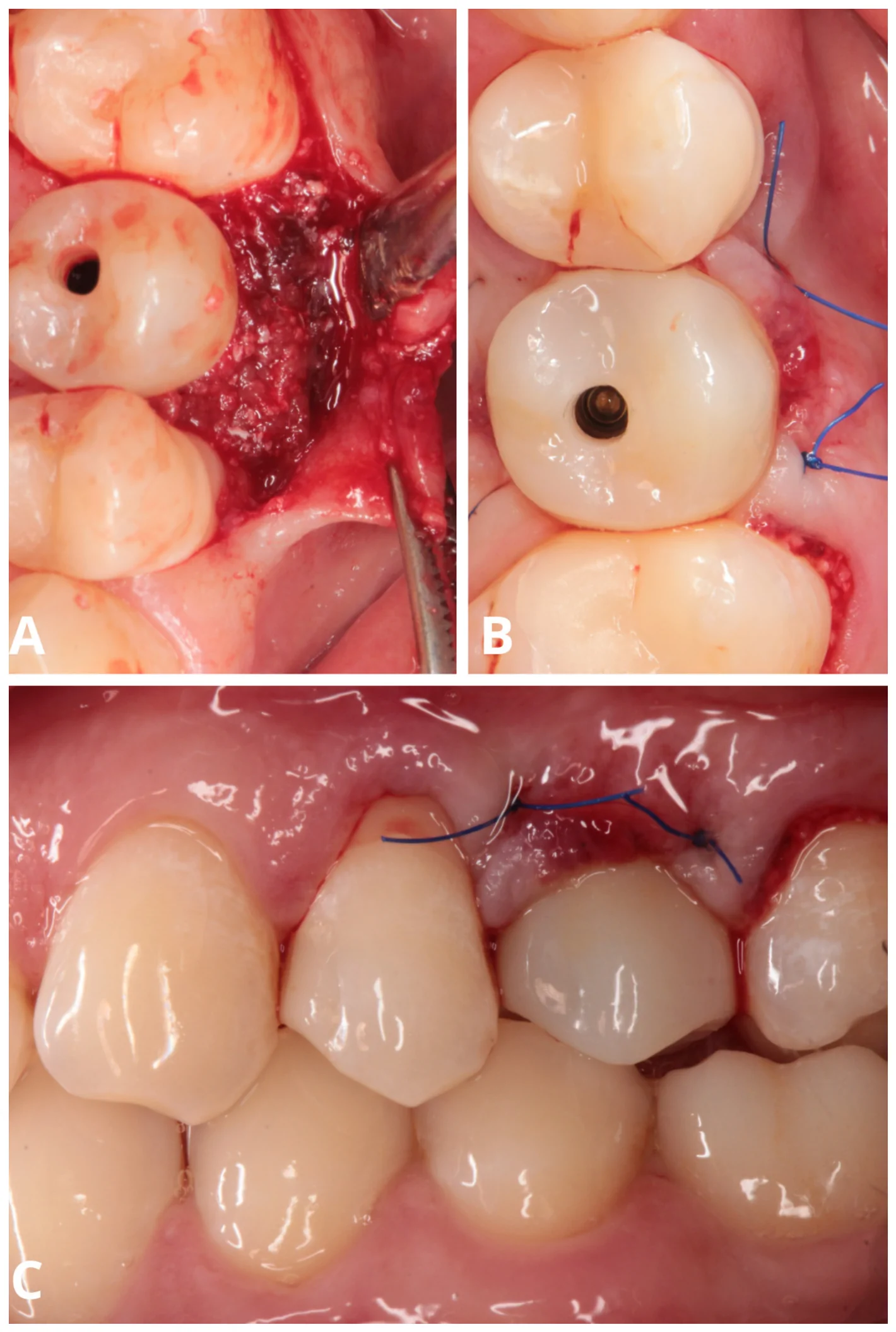
Placement of the provisional crown and closure of the surgical site: (**A**)—occlusal view of the provisional crown before suturing; (**B**)—occlusal view following suturing of the surgical site; (**C**)—lateral view of the surgical site following suturing.

A new provisional crown made of polymethyl methacrylate (PMMA) was placed on the implant, and the surgical site was closed using a 5-0 nonabsorbable monofilament polyamide suture (reference C0935123; Dafilon^®^; B. Braun Surgical S.A., Rubí, Spain), 75 cm in length, with a 16 mm reverse-cutting DS16 needle and a 3/8-circle curvature (Figure 8).

The provisional crown was adjusted to eliminate occlusal contact. Postoperative antibiotic therapy consisted of Augmentin 1000 mg administered every 12 h, with a total treatment duration of five days, including the day of surgery. Ibuprofen, a non-steroidal anti-inflammatory drug (NSAID), was prescribed for postoperative pain management as required. Nimesulide (Nimesil) was recommended as an alternative postoperative analgesic. Nimesulide is authorised in Poland for the short-term treatment of acute pain as a second-line therapy. It was selected because the patient had previously used Nimesil, reported good tolerability, and confirmed its effectiveness in relieving pain. Before prescribing the medication, the patient’s medical history, general health status, and relevant contraindications, including gastrointestinal conditions, were carefully assessed.

**Figure 9:**
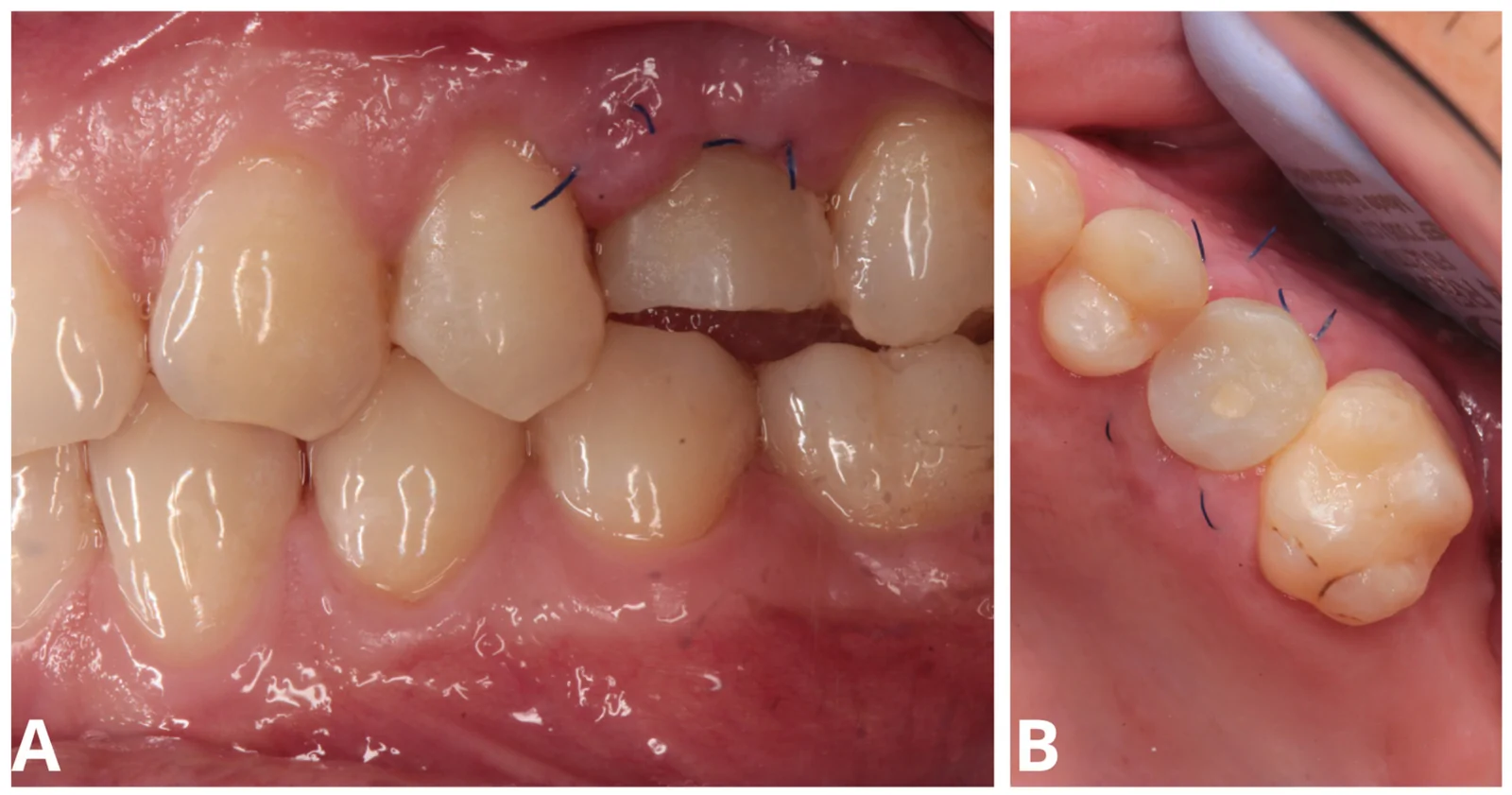
Condition of the surgical site one week postoperatively: (**A**)—lateral view; (**B**)—occlusal view.

One week after the procedure, the patient attended a follow-up visit for suture removal. The condition of the tissues was assessed as very satisfactory (Figure 9).

**Figure 10:**
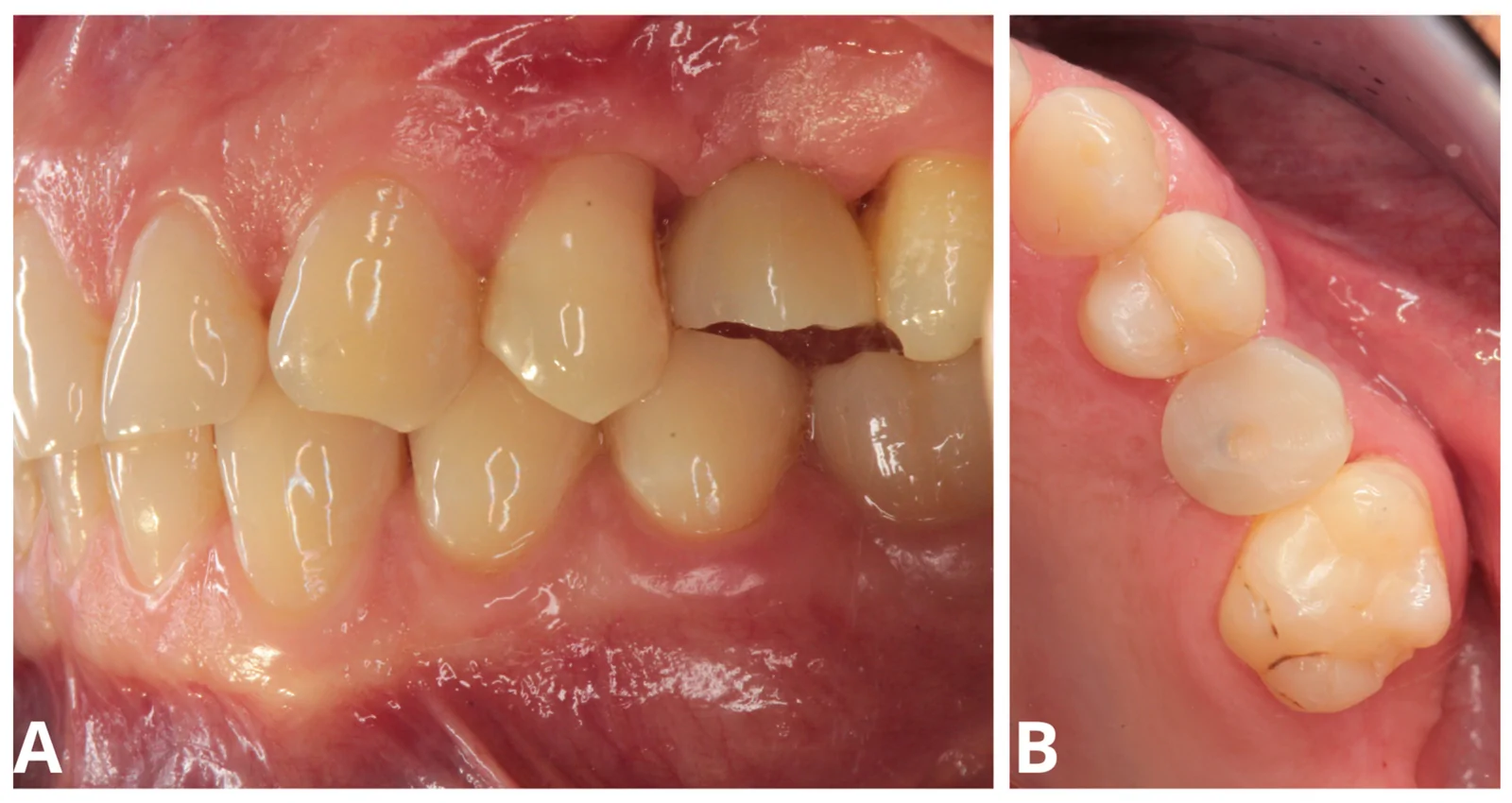
Condition of the tissues one month postoperatively: (**A**)—lateral view; (**B**)—occlusal view.

**Figure 11:**
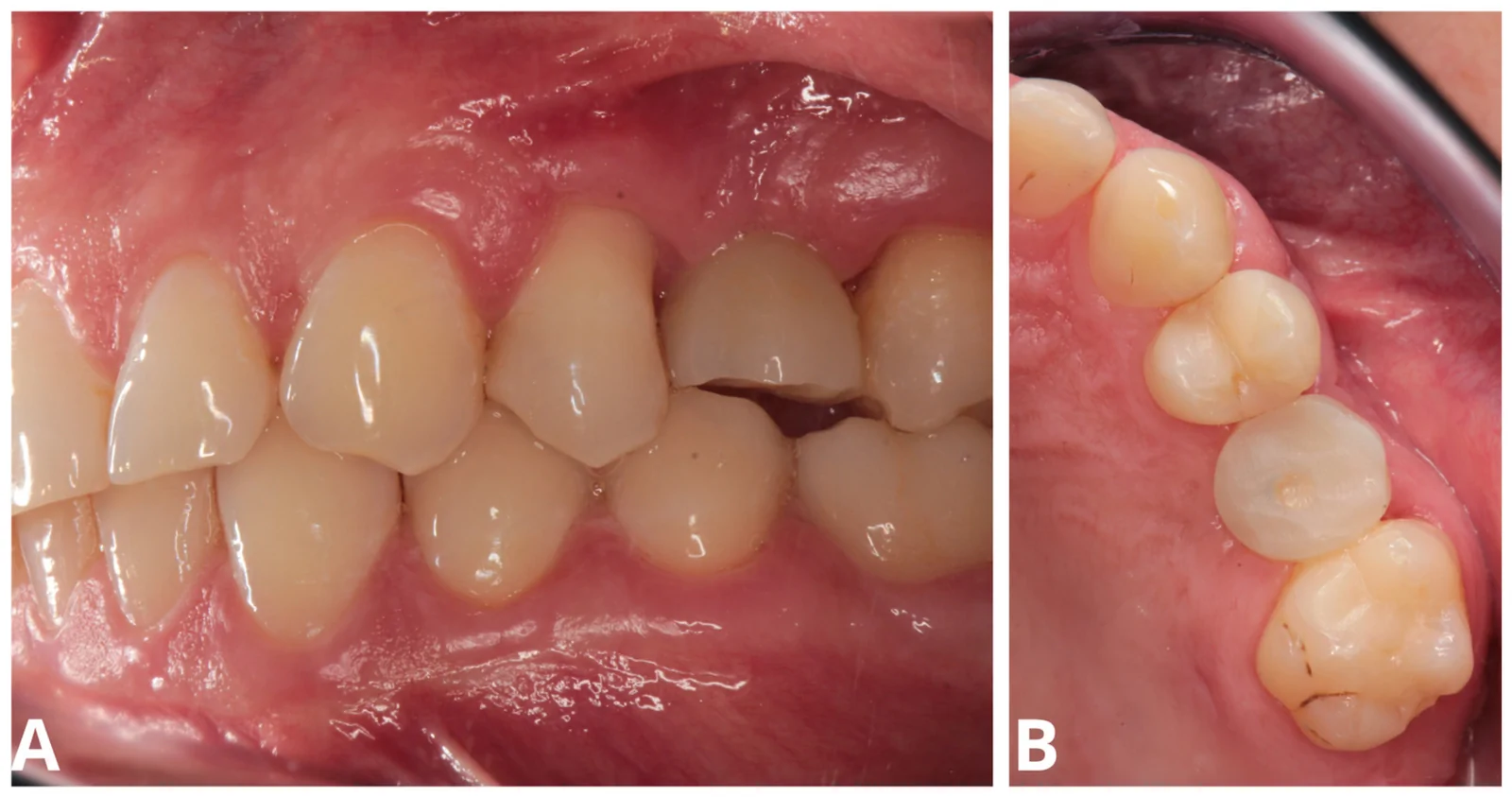
Condition of the tissues two months postoperatively: (**A**)—lateral view; (**B**)—occlusal view.

**Figure 12:**
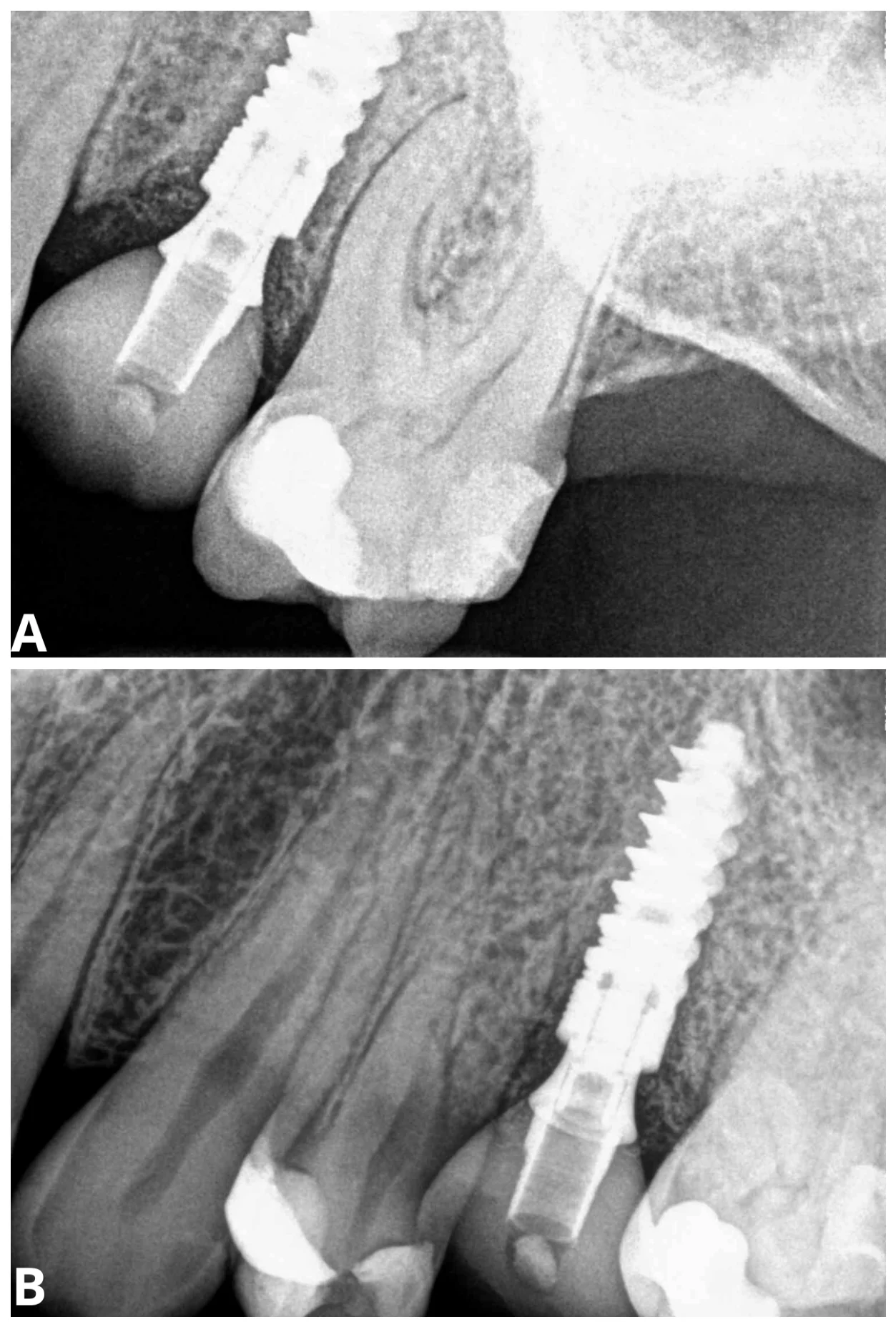
Radiographic follow-up: (**A**)—one month postoperatively; (**B**)—two months postoperatively.

Subsequent follow-up visits were scheduled at one month and two months postoperatively. The condition of the tissues remained satisfactory (Figure 10 and Figure 11), and the radiography images demonstrated increased radiopacity, indicating proper graft integration around the implant (Figure 12).

**Figure 13:**
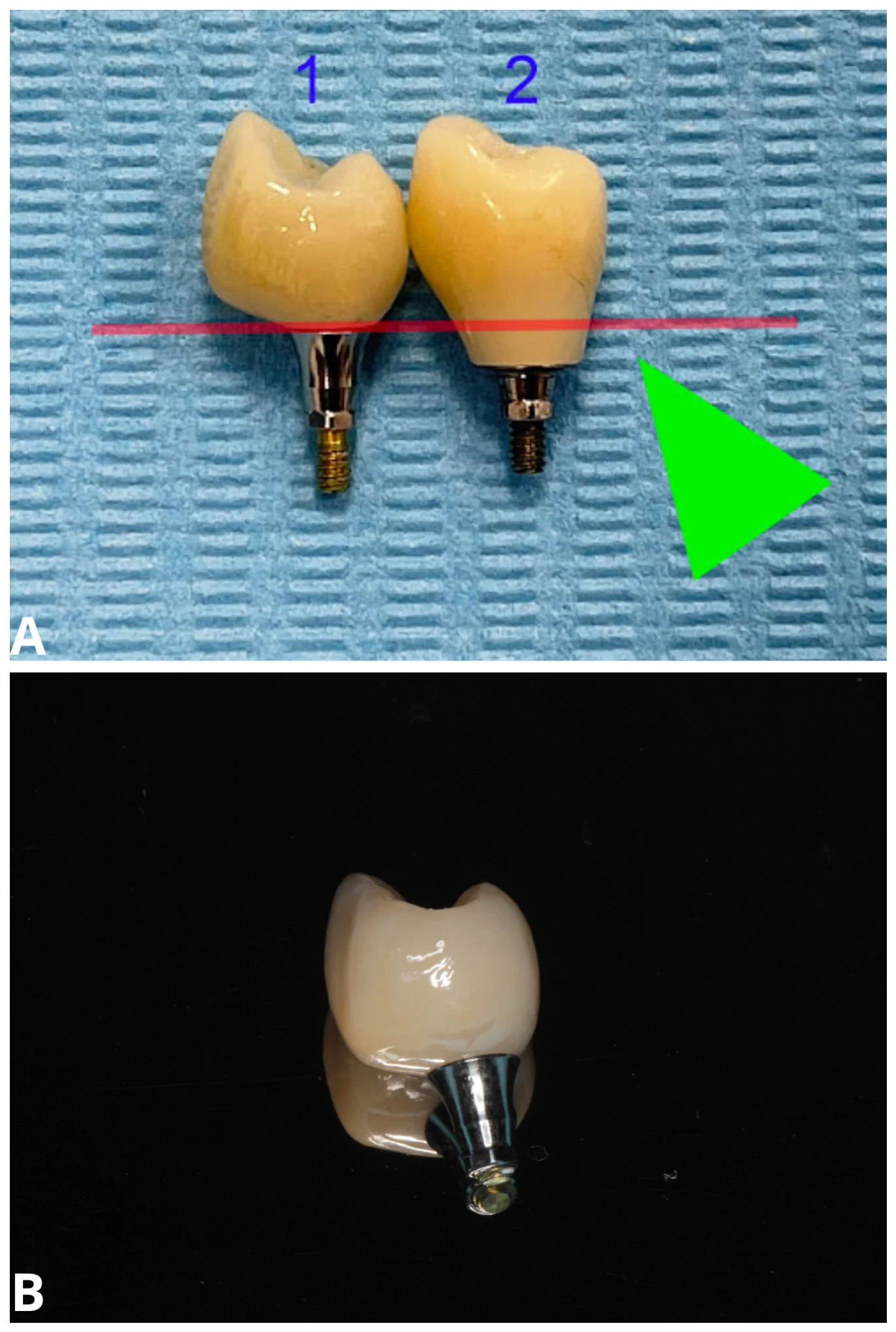
Comparison of the prosthetic crowns: (**A**)—(1) new crown and (2) previous crown, the green arrowhead indicates the cervical ceramic extension, and the red line serves as a reference line to illustrate the difference in its length; (**B**)—new definitive crown.

After four months, the implant at site 25 was restored with a definitive zirconia crown. At the design stage, it was decided to modify the emergence profile in order to reduce the risk of recurrent marginal bone loss (Figure 13). In the previous restoration, the ceramic material extended over a substantial part of the cervical region, creating a distinct step at the transition between the crown and the abutment. Such a profile may have limited access for oral hygiene procedures and increased plaque retention.

The definitive crown was placed, and the patient reported satisfaction with both the aesthetic and functional outcomes.

**Figure 14:**
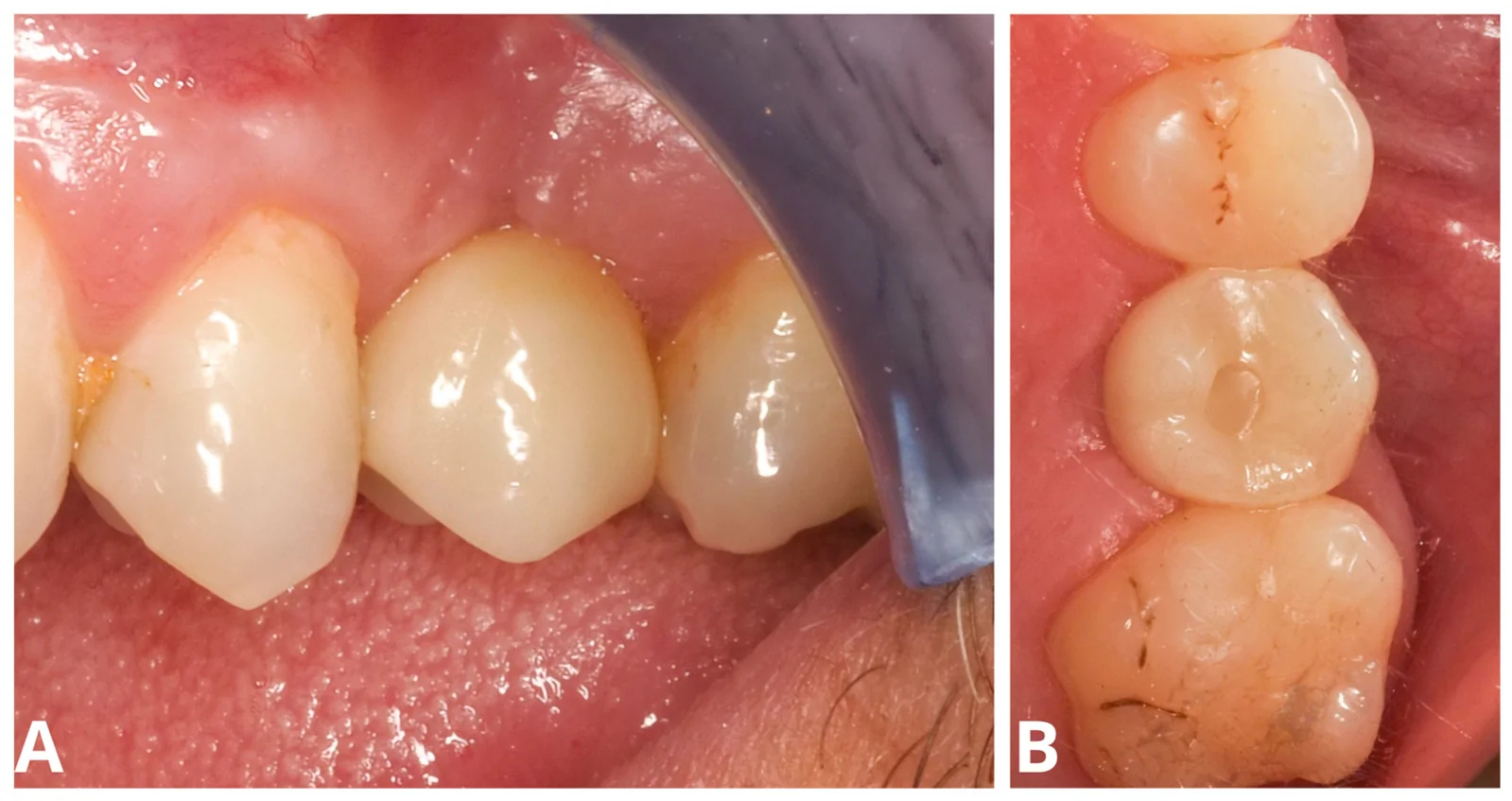
Two-year follow-up: (**A**)—lateral view; (**B**)—occlusal view.

**Figure 15:**
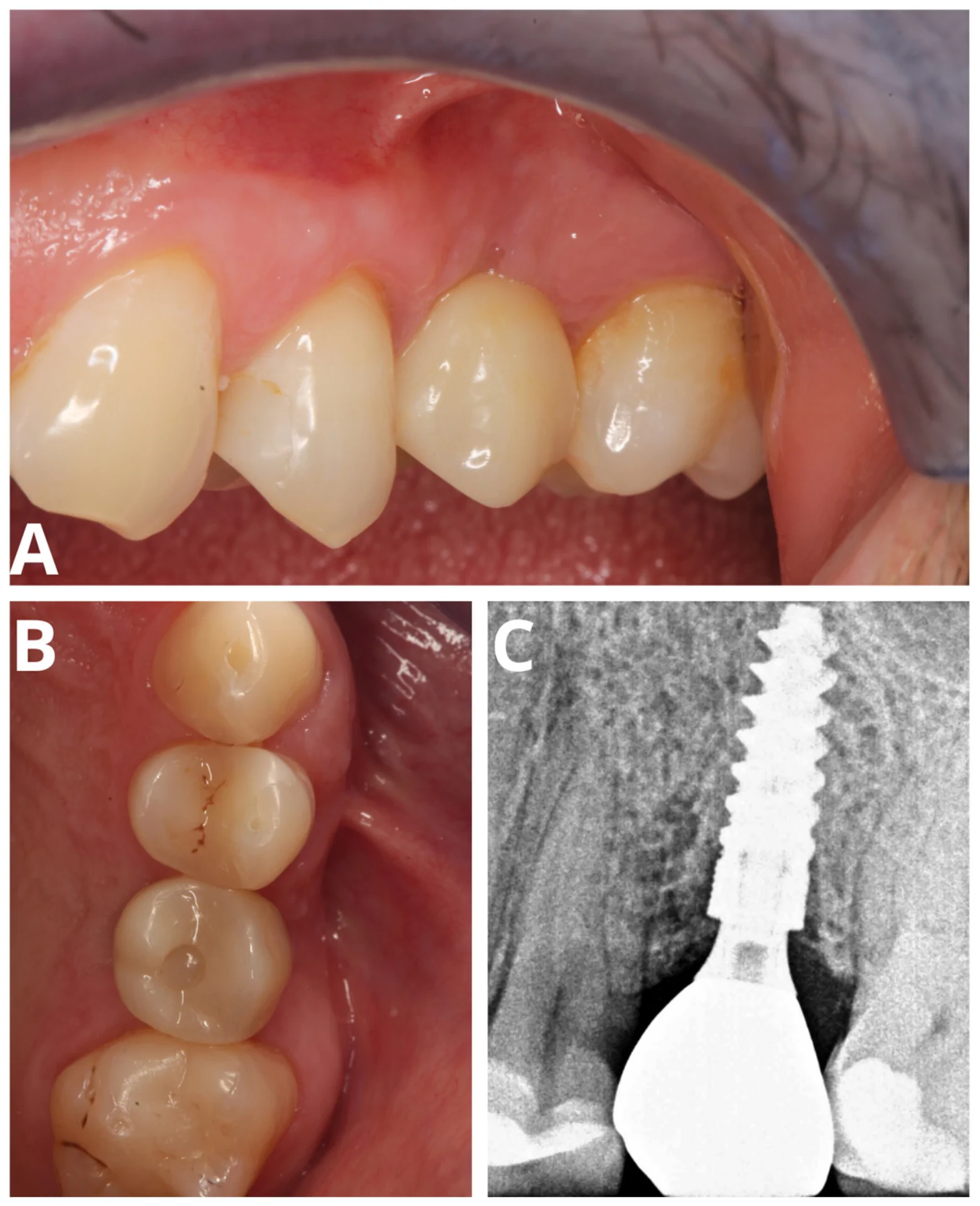
Three-year follow-up: (**A**)—lateral view; (**B**)—occlusal view; (**C**)—radiographic follow-up of the implant in position 25.

The patient remains under regular follow-up care. No symptoms have been reported, and imaging examinations demonstrate stable bone and soft tissue levels (Figure 14 and Figure 15). No bleeding on probing is observed, and the probing depth remains within the normal range, not exceeding 3.5–4 mm.

**Figure 16:**
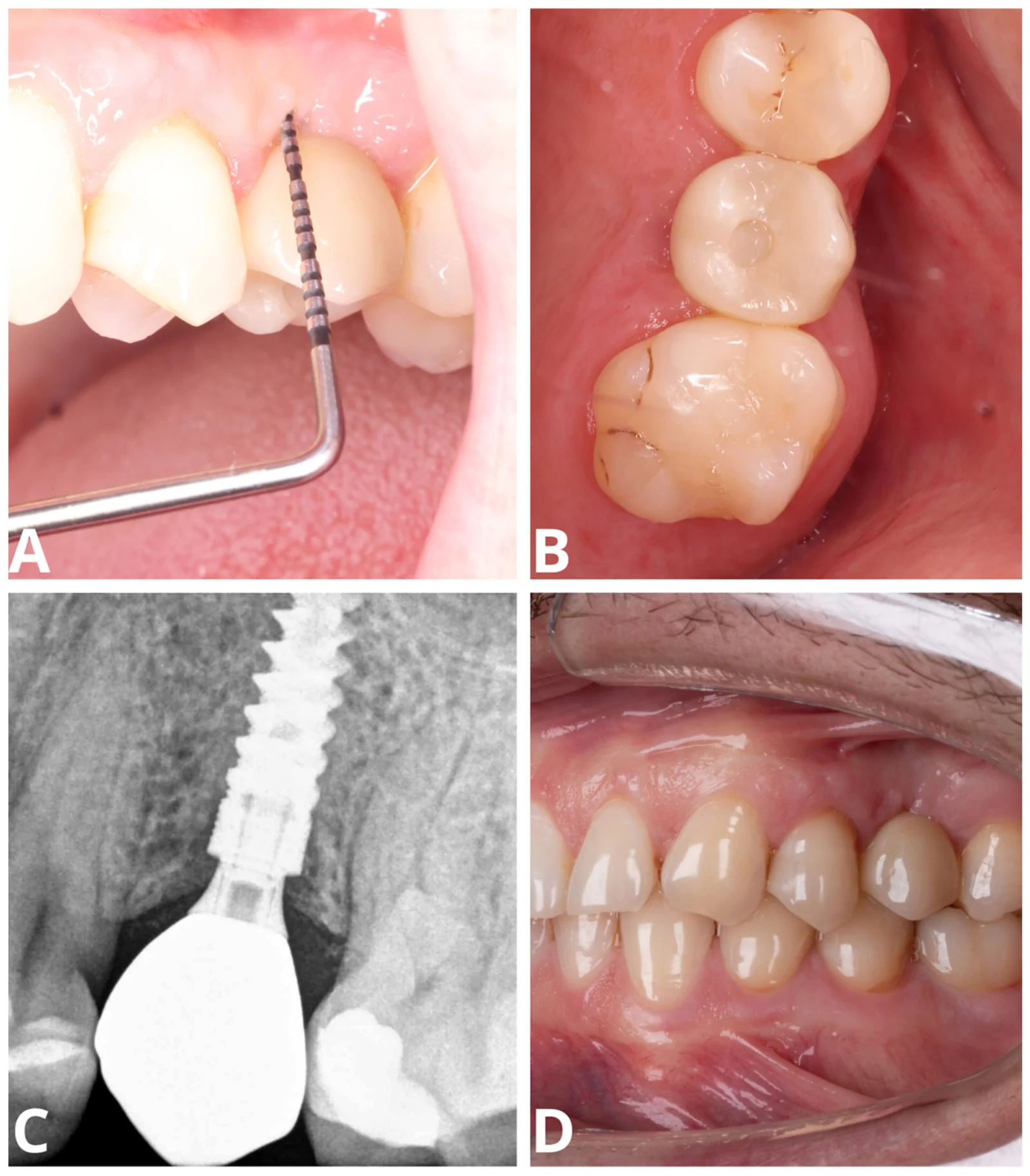
Five-year follow-up: (**A**)—probing of the treated site; (**B**)—occlusal view; (**C**)—radiographic follow-up of the implant at site 25; (**D**)—lateral view in occlusion.

**Figure 17:**
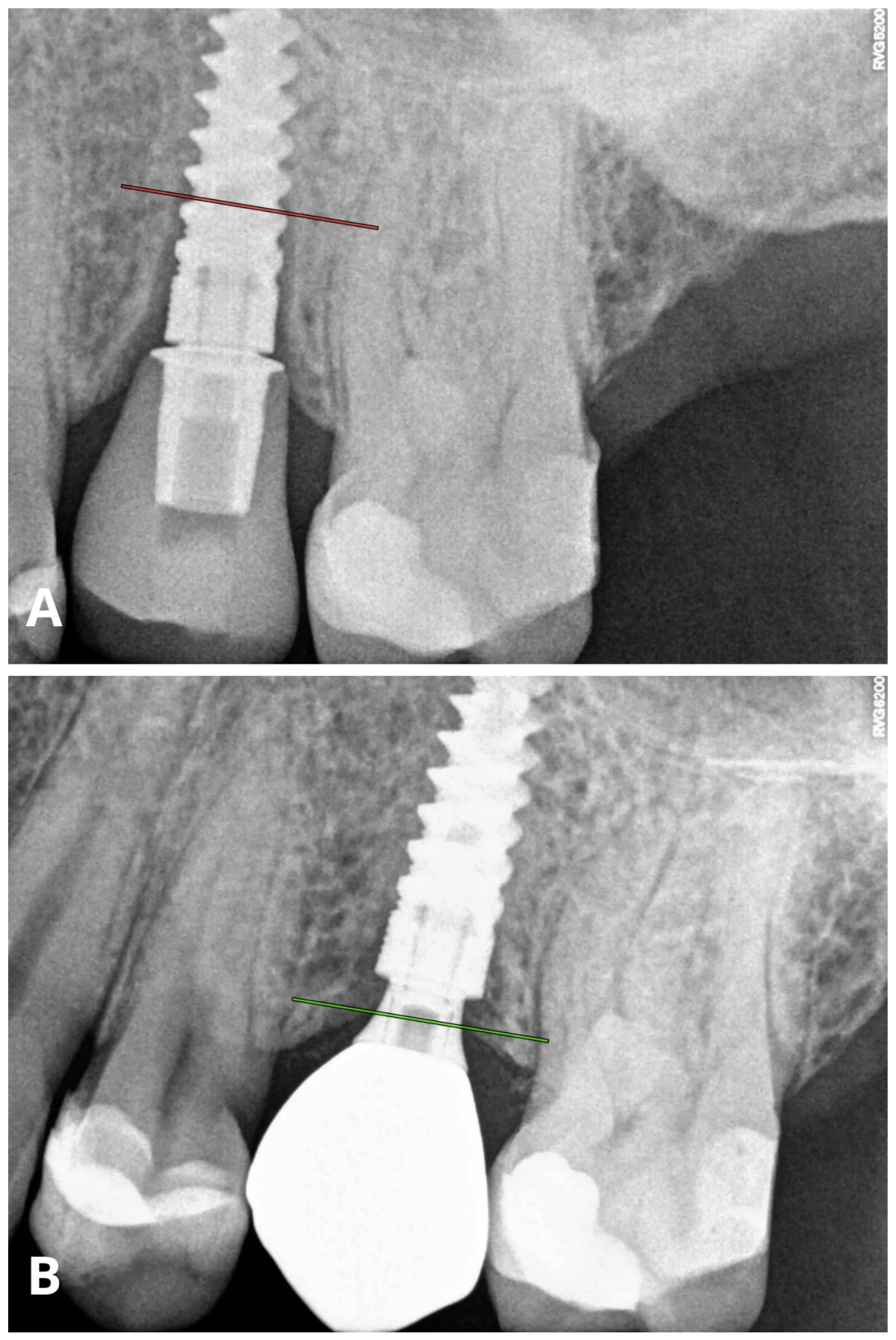
(**A**)—preoperative radiograph of the implant at site 25 with the level of the bone defect indicated (red line); (**B**)—5-year follow-up radiograph with the bone level indicated (green line).

At the five-year clinical follow-up, the treatment outcome remained stable, with no deterioration in aesthetic or functional conditions (Figure 16). Radiographic bone levels before treatment and at the five-year follow-up are shown in Figure 17. The patient continued to receive regular preventive care and maintained an individually tailored home oral hygiene regimen, including the use of Harmony Care mouthwash (Get Harmony Lab, Poznań, Poland) containing nanohydroxyapatite as an adjunct to mechanical biofilm control. The rationale for this approach is supported by laboratory findings demonstrating the antimicrobial and antibiofilm properties of the formulation, including its ability to reduce biofilm formation on titanium and zirconia surfaces used in implant–prosthetic treatment [23].

## 3. Discussion

Peri-implantitis is one of the most common peri-implant complications, affecting both soft and hard tissues and potentially leading to implant loss [24]. Long-term peri-implant bone stability is influenced by multiple patient-, implant-, site-, and prosthesis-related factors. Trombelli et al. [25] demonstrated the multifactorial nature of radiographic marginal bone changes around implants remaining in function for at least 5 years, emphasizing the importance of long-term clinical and radiographic monitoring and appropriate control of modifiable risk factors. In the present case, the absence of bleeding on probing, reduction in probing depth, and radiographic stability observed during the 5-year follow-up indicate a favourable long-term response to the combined surgical, regenerative, and prosthetic treatment.

Current evidence suggests that reconstructive surgical treatment may provide clinical and radiographic improvements in selected peri-implant defects. A network meta-analysis by Ramanauskaite et al. [26] indicated that guided bone regeneration using bone substitute materials may improve probing depth, bleeding outcomes, soft-tissue levels, and radiographic bone levels. However, the available evidence remains limited by the small number of comparative studies, methodological heterogeneity, and predominantly short follow-up periods. Furthermore, a recent systematic review of 17 long-term studies with follow-up periods of at least 5 years reported a mean disease resolution rate of 58.6%, a retreatment rate of 27.2%, and an implant survival rate of 88.6%. Due to substantial heterogeneity among the included studies, the results were summarised qualitatively and no meta-analysis was performed [27]. These findings indicate that implant survival should not be equated with complete disease resolution and highlight the importance of controlling residual inflammation and maintaining regular supportive care.

In their study, Sun et al. [28] evaluated the clinical effectiveness of platelet-rich fibrin (PRF) combined with guided bone regeneration (GBR) in the treatment of peri-implantitis-associated bone defects. The authors demonstrated that the combination of PRF, a bone substitute material, and GBR was associated with lower postoperative pain intensity, reduced bleeding, and greater density of the regenerated bone compared with flap surgery combined with GBR alone. These findings suggest that the use of PRF may favourably influence the healing process and support tissue regeneration around implants affected by inflammation. Similarly, the umbrella review by Acerra et al. [29] indicated that platelet concentrates, including PRF, may support wound healing and regenerative procedures in several fields of dentistry, including implant-related applications. Nevertheless, the authors also emphasized the heterogeneity of the available evidence and the need for further well-designed controlled clinical studies. This is consistent with the outcomes presented in the current report, in which surgical treatment combined with regenerative procedures resulted in improved bone conditions and stable soft tissues. However, because several therapeutic components were used simultaneously, the specific contribution of PRF to the observed outcome cannot be determined.

In the present case, the decision to preserve the implant and undertake regenerative treatment was of particular importance. The patient’s young age, good general health, and previously successful osseointegration of the implant, and the possibility of obtaining surgical access to the defect and modifying the prosthetic conditions supported the implementation of the described procedures. Implant preservation also avoided additional tissue trauma, prolonged treatment, and the potential need for further ridge augmentation associated with implant removal and subsequent replacement. Long-term implant retention, however, requires effective implant surface decontamination, resolution of inflammation, correction of local contributing factors, and regular supportive peri-implant care [30].

A prerequisite for successful regenerative treatment was thorough decontamination of the implant surface and removal of biofilm from the inflamed area, as the persistence of a contaminated surface could impair healing and reduce the predictability of tissue regeneration. Current systematic evidence has not demonstrated the clear superiority of any single implant-surface decontamination protocol [10]. Therefore, a combination of complementary techniques was used to facilitate the removal of granulation tissue, biofilm, and contaminants from the exposed implant surface. A similar combined clinical protocol was previously described by our group [31] and has subsequently been refined in clinical practice. The favourable result observed in the present case should not, however, be interpreted as evidence that this specific combination is superior to other available decontamination protocols.

Modification of the prosthetic restoration was another relevant component of treatment. The previous crown exhibited a cervical ceramic extension forming a distinct step at the crown–abutment junction, which could have impaired access for oral hygiene and promoted plaque retention. Previous studies have associated overcontoured implant-supported restorations, excessive emergence angles, and convex emergence profiles with plaque accumulation, peri-implant inflammation, and a higher prevalence of peri-implantitis [32,33,34]. The new definitive zirconia crown was therefore designed with a modified cervical contour to improve accessibility for plaque control and facilitate professional maintenance. Although the independent effect of the prosthetic modification cannot be determined in a single case, elimination of the plaque-retentive contour may have contributed to the stable soft-tissue conditions and favourable long-term outcome. Bone regeneration alone, without correction of prosthetic factors promoting biofilm retention, might not have provided an equally stable result.

The findings of the present report are also consistent with previously published clinical cases describing favourable long-term outcomes after reconstructive peri-implantitis treatment. Etemadi et al. [35] reported a 5-year outcome after surgical debridement, chemical decontamination, adjunctive antimicrobial photodynamic therapy, xenogeneic bone grafting, and placement of a resorbable collagen membrane. At the final examination, reduced probing depths, complete intrabony defect fill, and stable bone levels were observed. Khetarpal et al. [36] presented a staged approach combining guided bone regeneration with subsequent soft-tissue augmentation and reported stable peri-implant hard- and soft-tissue conditions after 5 years. Bassi et al. [37] reported successful implant preservation 17 years after surgical peri-implantitis treatment, with physiological probing depths, no clinical signs of peri-implant inflammation or bleeding on probing, and no further radiographic bone loss.

These reports share several features with the present case, including surgical access, thorough implant-surface decontamination, the use of regenerative materials, correction of local risk factors, and long-term maintenance. However, they differ in the decontamination methods, grafting materials, use of barrier membranes or soft-tissue augmentation, and the number and sequence of surgical interventions. In the present case, the treatment protocol combined mechanical implant-surface decontamination, regenerative therapy using a bone substitute and platelet concentrates, and modification of the prosthetic restoration. The 5-year clinical and radiographic stability observed in this case supports the potential value of addressing the biological, regenerative, and prosthetic aspects of peri-implantitis within a comprehensive treatment strategy.

Resonance frequency analysis and implant stability quotient (ISQ) measurements were not included in the treatment protocol at the time of the procedure, as these methods were not routinely used in our clinical practice during that period. Consequently, these data cannot be obtained retrospectively. Although the implant remained clinically stable and the treatment outcome was supported by clinical and radiographic findings, serial ISQ measurements could have provided an additional non-invasive and objective parameter for monitoring implant stability at different stages of treatment and follow-up [38]. The incorporation of resonance frequency analysis may therefore be considered in future clinical cases and prospective studies assessing regenerative peri-implantitis treatment.

Several limitations should be acknowledged. First, this report describes a single clinical case; therefore, the observed outcome cannot be generalised or used to determine the relative contribution of each component of the combined treatment protocol. Furthermore, the patient was young, systemically healthy, and had no relevant comorbidities or regular medication use. These favourable conditions may have positively influenced tissue healing and regenerative potential, and comparable outcomes may not necessarily be achieved in patients with systemic diseases, impaired immune responses, or additional risk factors. Probing depths were recorded at four rather than six peri-implant sites, and standardised numerical plaque and bleeding indices were not documented. Serial ISQ measurements were also unavailable. Finally, the simultaneous use of several decontamination, regenerative, and prosthetic interventions prevents conclusions regarding the individual effectiveness of any single component. Further prospective studies using standardised clinical indices, objective implant stability measurements, clearly defined defect morphology, and long-term follow-up are required to determine the predictability of comparable combined treatment protocols.

## 4. Conclusions

The combination of surgical decontamination, regenerative treatment using PRF and sticky bone, and modification of the crown emergence profile resulted in a stable clinical and radiographic outcome over a five-year follow-up period.

Early identification and correction of the factors contributing to the development of peri-implantitis were of key importance. In carefully selected cases with a similar clinical presentation and aetiology, the protocol described in this report may be considered as a comprehensive therapeutic approach. However, further clinical studies are required before its predictability and broader applicability can be established.

## 5. Patient Perspective

The patient reported satisfaction with the treatment outcome and did not report any symptoms or discomfort.

## Figures and Tables

**Table 1:** Sequence of treatment stages.

Description	Treatment Stage
Clinical and radiographic examination revealed peri-implant bone loss around the implant in position 25, bleeding on probing, and a probing depth of 7 mm	Initial follow-up visit
Decision to preserve the implant, perform surgical decontamination and bone augmentation, and replace the existing prosthetic crown	Treatment planning
Removal of the prosthetic crown and elevation of a mucoperiosteal flap to expose the peri-implant bone defect; mechanical and laser-assisted decontamination; bone augmentation	Surgical access, implant decontamination and regenerative procedure
Placement of a provisional crown without occlusal contact	Provisional restoration
Suture removal after one week and clinical follow-up at one and two months, demonstrating satisfactory soft tissue healing and progressive graft integration	Early healing phase
Placement of the definitive crown after four months, with a modified emergence profile	Definitive prosthetic rehabilitation
Clinical and radiographic monitoring over five years, demonstrating stable bone and soft tissue levels	Long-term follow-up

## Data Availability

The data that support the findings of this study are available from the corresponding author upon reasonable request.
